# Prenatal Alcohol Exposure and Congenital Heart Defects: A Meta-Analysis

**DOI:** 10.1371/journal.pone.0130681

**Published:** 2015-06-25

**Authors:** Jiaomei Yang, Huizhen Qiu, Pengfei Qu, Ruo Zhang, Lingxia Zeng, Hong Yan

**Affiliations:** 1 Department of Epidemiology and Health Statistics, School of Public Health, Xi’an Jiaotong University Health Science Center, Xi’an, Shaanxi, People’s Republic of China; 2 Nutrition and Food Safety Engineering Research Center of Shaanxi Province, Xi’an, Shaanxi, People’s Republic of China; Tabriz University of Medical Sciences, ISLAMIC REPUBLIC OF IRAN

## Abstract

**Background:**

There are still inconsistent conclusions about the association of prenatal alcohol drinking with congenital heart defects (CHDs). We conducted this meta-analysis to investigate the association between prenatal alcohol exposure and the risk of overall CHDs and the CHDs subtypes.

**Methods:**

Case-control and cohort studies published before March 2015 were searched through PubMed and Embase. Two authors independently extracted data and scored the study quality according to the Newcastle-0ttawa Scale. The pooled ORs and 95%CI were estimated using the random-effects model and heterogeneity was assessed by the Q test and I^2^ statistic.

**Results:**

A total of 20 studies were finally included. The results provided no evidence of the association between prenatal alcohol exposure and the risk of overall CHDs (OR = 1.06, 95%CI = 0.93–1.22), ventricular septal defects (VSDs) (OR = 1.04, 95%CI = 0.86–1.25), or atrial septal defects (ASDs) (OR = 1.40, 95%CI = 0.88–2.23). However, prenatal alcohol drinking was marginally significantly associated with conotruncal defects (CTDs) (OR = 1.24, 95%CI = 0.97–1.59) and statistically significantly associated with d-Transposition of the Great Arteries (dTGA) (OR = 1.64, 95%CI = 1.17–2.30). Moreover, both prenatal heavy drinking and binge drinking have a strong association with overall CHDs (heavy drinking: OR = 3.76, 95%CI = 1.00–14.10; binge drinking: OR = 2.49, 95%CI = 1.04–5.97), and prenatal moderate drinking has a modest association with CTDs (OR = 1.35, 95%CI = 1.05–1.75) and dTGA (OR = 1.86, 95%CI = 1.09–3.20).

**Conclusions:**

In conclusion, the results suggested that prenatal alcohol exposure was not associated with overall CHDs or some subtypes, whereas marginally significant association was found for CTDs and statistically significant association was found for dTGA. Further prospective studies with large population and better designs are needed to explore the association of prenatal alcohol exposure with CHDs including the subtypes in specific groups.

## Introduction

Congenital heart defects (CHDs) refer to the structural anomalies of the heart and great vessels that are present at birth and can disrupt the normal flow of blood through the heart or vessels near it. As the most prevalent congenital abnormalities in the world, CHDs account for nearly thirty percent of total major congenital anomalies[1], with an estimated birth prevalence of 9.1 in 1,000 live births[2]. Moreover, CHDs are the leading cause of infant morbidity and death from birth defects [3]. Those survived children with CHDs may undergo physical, developmental, or cognitive problems [4, 5], which requires special medical treatments and increases the burdens of family and society. The etiology for cases with CHDs is largely unknown, but several genetic anomalies, some maternal illnesses, and prenatal exposures to specific therapeutic and non-therapeutic drug are generally accepted as risk factors[6].

Prenatal alcohol exposure can exert a wide range of adverse effects on the developing fetus, such as craniofacial abnormalities, growth deficiencies, central nervous systems dysfunctions, and neurobehavioral disabilities, collectively known as fetal alcohol spectrum disorders (FASD). Animal studies have indicated that maternal exposure to alcohol during the gestational period increases the incidence of heart anomalies in the offspring [7, 8]. According to one review that summarized studies published before 2007, the proportion of CHDs in infants with FASD accounted for 67% [9]. The possible teratogenic effects depend on the timing, frequency, duration, amount of alcohol exposure as well as genetic susceptibility[10]. Despite the risks, many women still drink in pregnancy, 34.8% in Australia[11] and 30.3% in America[12].

Although the effect of maternal alcohol consumption on CHDs has been explored for decades, there are still inconsistent conclusions about the association of maternal alcohol exposure with CHDs, including the CHDs subtypes. To our knowledge, there has been no comprehensive meta-analysis to explore the relationship between prenatal alcohol exposure and CHDs. Two previous systematic reviews written by Henderson et al. [13, 14] tried to estimate the effects of prenatal low-to-moderate and binge drinking on pregnancy outcomes, including birth defects. However, both studies were not able to carry out meta-analyses because of the high heterogeneity in the methods of included articles and they did not separately consider CHDs, the most common group of birth defects. The aim of this meta-analysis was to investigate the association between prenatal alcohol exposure and the risk of overall CHDs as well as the CHDs subtypes.

## Methods

### Search strategy

We performed the meta-analysis and reported the results in accordance with the Preferred Reporting Items for Systematic Reviews and Meta-Analyses (PRISMA) guidelines (**S1 Checklist**). The protocol of this meta-analysis was not registered anywhere. We searched PubMed and Embase to identify all relevant case-control and cohort studies published as original articles in English up to March 2015. The following medical subject headings and text words were used to identify relevant articles: (“drinking” or “drinking behaviors” or “alcohol drinking” or “alcohol consumption” or “alcohol exposure” or “alcoholic beverages” or “alcohol”) AND (“congenital heart defects” or “cardiovascular abnormalities” or “cardiovascular diseases” or “congenital abnormalities” or “birth defects” or “congenital malformations”). We also scanned the reference lists of the identified articles to find additional publications of interest.

### Eligibility criteria

Two reviewers independently scanned all the titles and abstracts of the retrieved studies to exclude distinctly irrelevant ones, and then they independently assessed the full texts to determine whether the remaining articles met the eligibility criteria. Discrepancies between the two reviewers during each stage were settled by discussion with the third reviewer. A published study was included if it met the following inclusion criteria:

1. cohort or case-control study that investigated the relationship between maternal alcohol exposure before or during pregnancy and the risk of overall CHDs or any CHDs subtypes;
2. relative risk (RR) or odds ratios (ORs) and the corresponding 95% confidence intervals (CIs) could be directly extracted or calculated from the available data;
3. original study without duplicated data.

We didn’t include studies where mothers of interest were diagnosed with CHDs, diabetes, or other abnormal conditions. We also exclude studies where the infants of interest were specifically referred to those with Down syndrome. Animal studies, letters, editorials, conference abstracts, reviews, or comments were not included. If there were multiple articles with the same or overlapped data, only the study with the most comprehensive information, such as the longest study period or the most overall CHD subtypes, was selected.

### Data extraction and Study quality assessment

Data extraction and study quality assessment were performed independently by two reviewers through a piloted form, and any disagreement was resolved by discussion with the third reviewer. The following information were recorded from the eligible studies: first author’s surname, publication year, study period, study type (cohort, population-based case-control, and hospital-based case-control), study country, sample size, ascertainment of alcohol exposure(in-person interview, telephone interview, email, medical records, and birth records) and CHDs (clinical examination, echocardiography, cardiac catheterization, cardiac surgery, autopsy, medical records, birth certificate, and birth defects registers), timing of drinking, adjusted or matched confounders, risk estimates and corresponding 95% CIs. The methodological quality of the included studies was scored according to the Newcastle-0ttawa Quality Assessment Scale (NOS)[15]. This scale assesses the selection of the study sample, the comparability of the study groups, and the ascertainment of either the exposure or outcome for case-control or cohort studies respectively, with a highest total score of nine points. A score of ≥6 was used to distinguish high-quality from poor-quality studies.

### Statistical analyses

The measure of interest was OR. We used the adjusted OR that was controlled for potential confounders in the greatest degree whenever available; otherwise, we utilized the unadjusted OR or computed it from the exposure distributions given in the studies. A random-effects model was applied to estimate the pooled ORs and 95%CI[16] because of anticipated heterogeneity across the included studies. For the studies with dose-response relationship, we transformed alcohol consumption categories into grams of absolute alcohol per day as the common unit of measure. One standard drink was defined as 12g of pure alcohol if there was no report about grams of alcohol per drink in the study [17]. The nondrinking group was chosen as the reference category. Binge drinking was defined as consumption of ≥48g alcohol on one or more occasion. Heavy drinking was characterized as consumption of ≥24g alcohol per day, and moderate drinking as consumption of < 24g alcohol per day. Statistical heterogeneity across studies was assessed by both the Cochrane’s Q-test and I^2^ statistic[18]. *P*<0.10 or I^2^ >50% was suggestive of statistically significant heterogeneity. Moreover, we performed subgroup analyses according to geographical area (North America, Europe, and Australia), study type, timing of drinking, publication year (before or in 2000, and after 2000), and sample size (>1000, and ≤1000). We carried out sensitivity analysis by reanalyzing the summary estimates after removal of each study in turn. We also conducted sensitivity analysis by restricting the analysis to high-quality studies. Publication bias was assessed by funnel plot and Egger’s regression asymmetry test[19] when adequate numbers of studies were included in the analyses. *P*<0.05 was considered to be statistically significant. Analyses were conducted using STATA software (version 12; StataCorp, College Station, Texas, USA).

## Results

### Literature search

**Fig 1:**
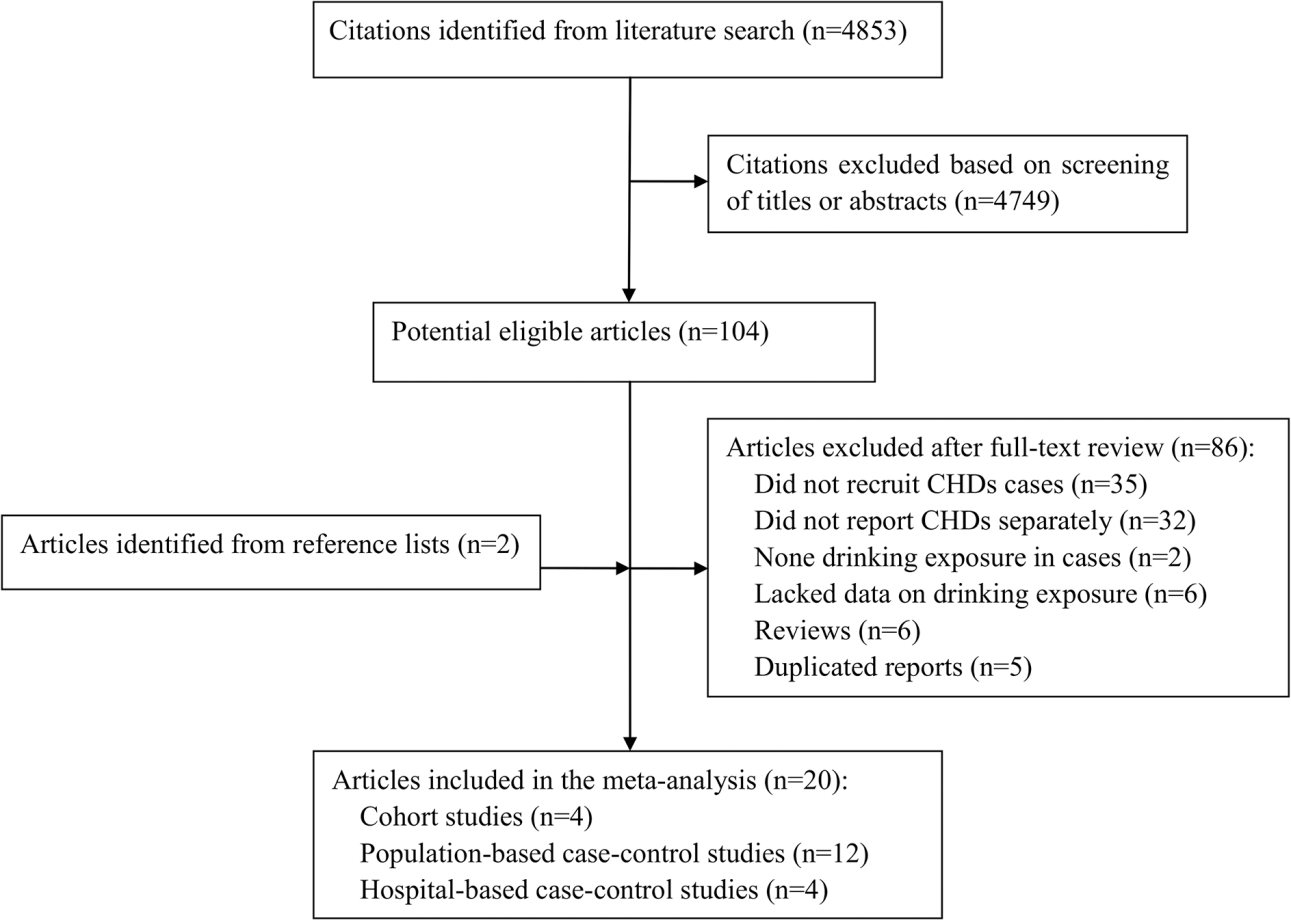
Flow chart of screening for literature about prenatal alcohol exposure and congenital heart defects.

The flow chart of literature search and study selection is shown in **Fig 1**. From the 4853 citations retrieved in the electronic databases, 104 had the potential to be eligible after the initial screening based on titles or abstracts. Of these, 86 articles were excluded after further reviewing the full texts, leaving 18 studies for inclusion. Additionally, two eligible articles were identified from the reference lists. Thus, a total of 20 studies were found to meet all the inclusion criteria for final inclusion in the meta-analysis.

### Study characteristics

The characteristics of the included studies are shown in **S1 Table**. The 20 studies were published between 1987 and 2013 and the study period ranged from 1974 to 2010, with a total population of 310,919. Of these studies, four were cohort studies [20–23], 12 were population-based case-control studies [24–35], and four were hospital-based case-control studies [36–39]. Nine were conducted in America, one in Canada, one in Australia, and nine in European countries. Three articles [25–27] conducted in Finland were actually from the same population-based case-control design during 1982–1983, but they displayed different outcomes of interest. The NOS score ranged from 4 to 8, with a median of 6. Score below 6 tended to arise from failure to adjust for confounders, getting maternal alcohol exposure without blinded to the interviewer, and failure to collect cases with CHDs from stillbirth or pregnancy loss.

### Prenatal alcohol drinking vs. none drinking and CHDs risk

#### Overall CHDs

**Fig 2:**
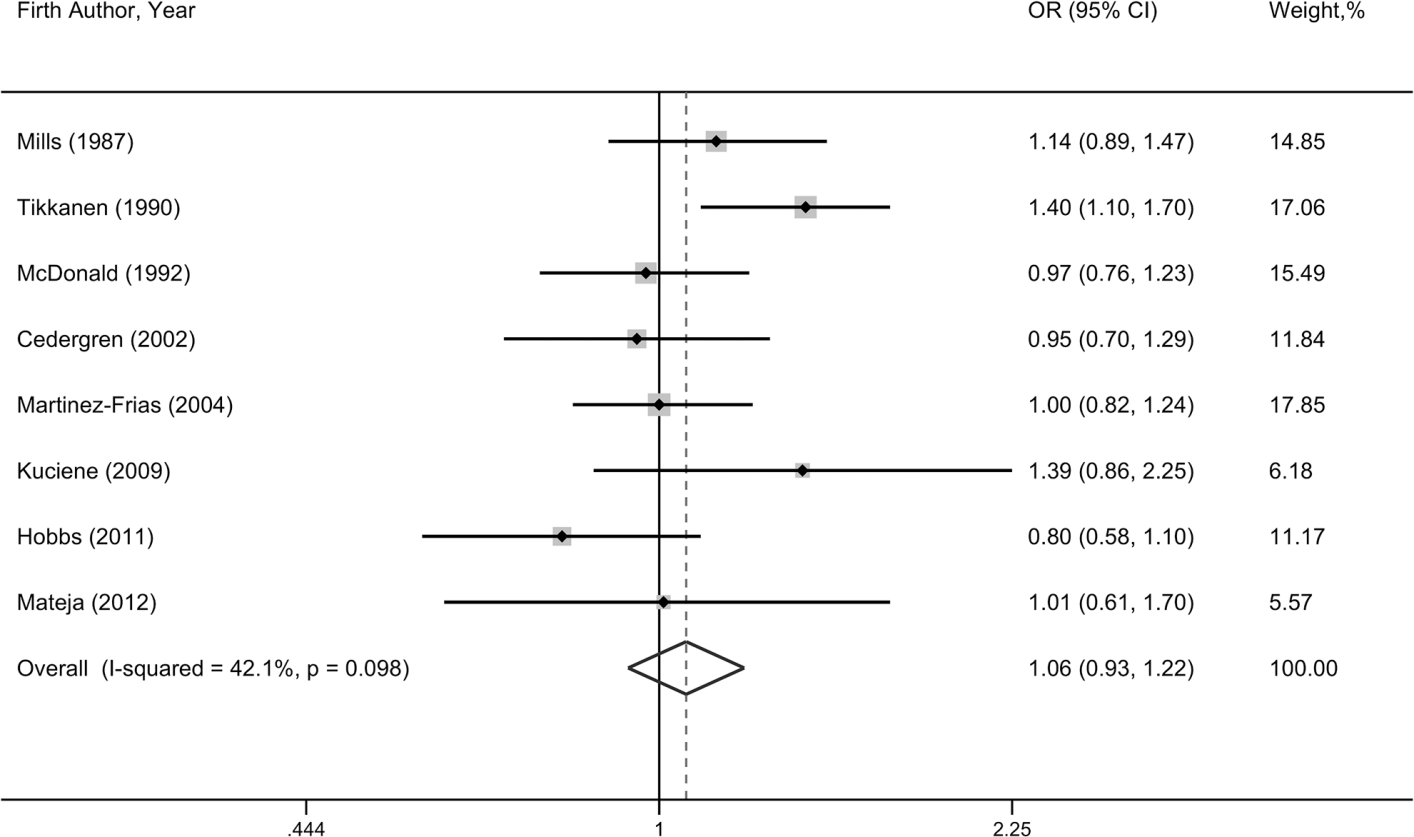
Forest plot of any prenatal alcohol exposure and overall congenital heart defects risk.

The relationship between prenatal alcohol exposure and overall CHDs was investigated in 8 studies, with a total number of 3,749 cases and 122,200 controls. The pooled result suggested that prenatal alcohol drinking seemed to slightly increase overall CHDs risk, but the confidence interval included unity (OR = 1.06, 95%CI = 0.93–1.22) (**Fig 2**). No significant heterogeneity among studies was found (*P* = 0.10, I^2^ = 42.1%). Subgroup analyses stratified by geographical area, study type, timing of drinking, or publication year yielded no significant association (**Table 1**). However, a borderline significantly higher risk of overall CHDs associated with prenatal alcohol intake was found in studies with sample size larger than 1,000 (OR = 1.11, 95%CI = 0.96–1.29).

**Table 1:** Summary results of the association between prenatal alcohol exposure and overall congenital heart defects risk.

Group	No. of studies	OR(95%CI)	*P* for heterogeneity	I^2^ (%)
Total	8	1.06(0.93–1.22)	0.10	42.1
High-quality studies ^a^	3	1.11(0.88–1.40)	0.04	69.5
Geographical area				
North America	4	0.99(0.85–1.14)	0.40	0
Europe	4	1.22(0.93–1.60)	0.07	57
Study type				
cohort	2	1.05(0.88–1.25)	0.36	0
population-based case control	5	1.08(0.85–1.39)	0.04	61.3
hospital-based case control	1	1.00(0.81–1.23)	-	-
Timing of drinking				
first trimester	4	1.12(0.93–1.34)	0.09	53.9
during pregnancy	2	1.09(0.82–1.46)	0.22	34.2
periconception	1	0.80(0.58–1.10)	-	-
before pregnancy	1	1.01(0.61–1.69)	-	-
Publication year				
≤ 2000	3	1.16(0.94–1.44)	0.08	59.8
> 2000	5	0.98(0.85–1.12)	0.45	0
Sample size				
≤ 1000	3	0.97(0.74–1.28)	0.17	43.1
> 1000	5	1.11(0.96–1.29)	0.15	40.5

^a^ Studies scoring 6 points or higher were considered as high quality, and those scoring lower than 6 points as low quality.

#### Ventricular septal defects

**Fig 3:**
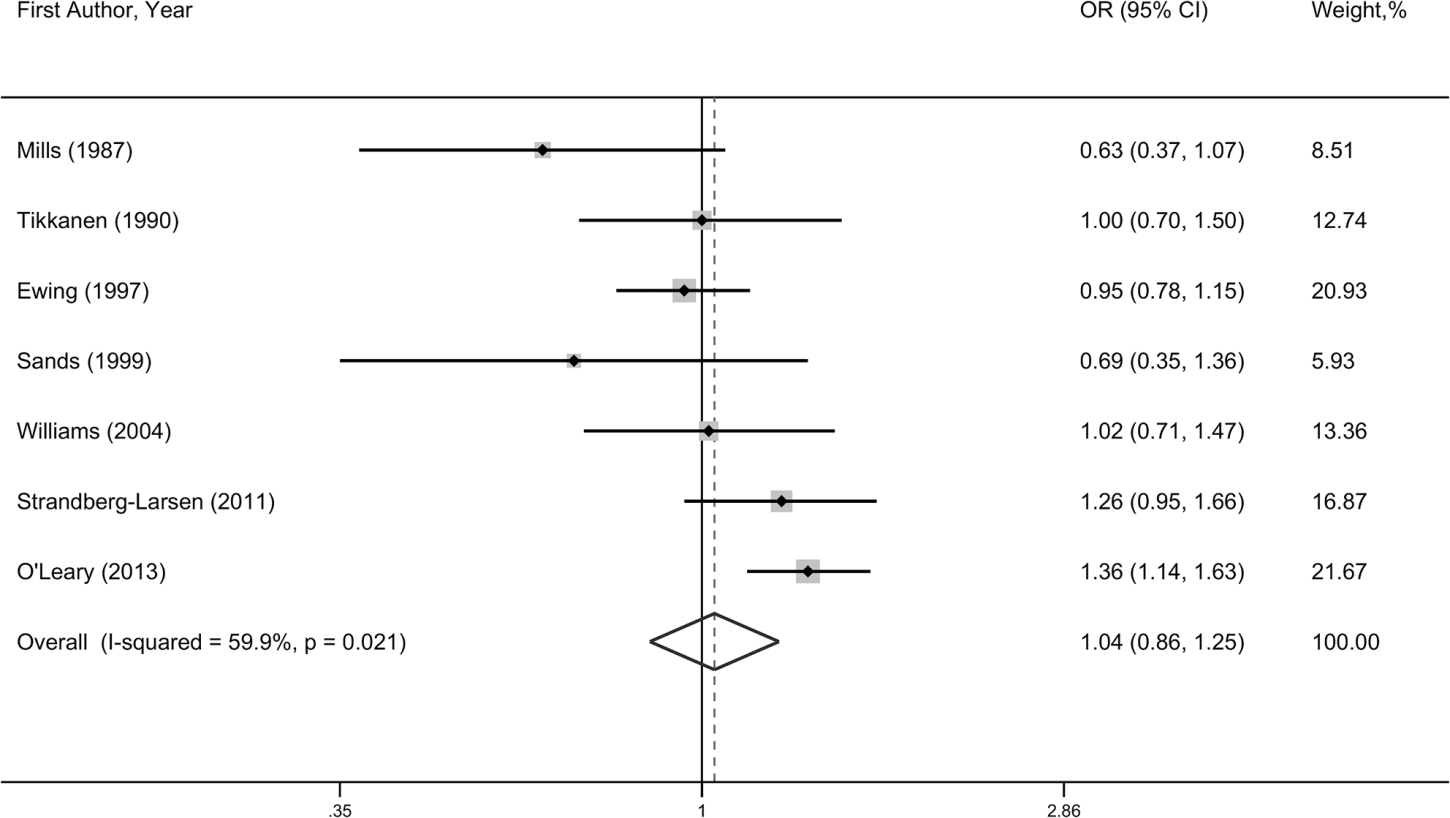
Forest plot of any prenatal alcohol exposure and ventricular septal defects risk.

The association between prenatal alcohol exposure and ventricular septal defects (VSDs) was assessed in seven studies, with a total number of 1,719 cases and 198,285 controls. No significant difference in VSD risk was found between mothers who drank before or during pregnancy and those who did not drink (OR = 1.04, 95%CI = 0.86–1.25), and significant heterogeneity was observed (*P* = 0.02, I^2^ = 59.9%) (**Fig 3**). Subgroup analysis by geographical area showed the same nonsignificant association in North America (OR = 0.93, 95%CI = 0.79–1.09) and Europe (OR = 1.10, 95%CI = 0.89–1.63), while one study conducted in Australia reported an increased risk of VSD (OR = 1.36, 95%CI = 1.14–1.63). Moreover, subgroup analysis indicated a borderline significant association among studies that explored maternal alcohol drinking during pregnancy (OR = 1.23, 95%CI = 0.97–1.55) and a significant association among studies published in 2000 later (OR = 1.28, 95%CI = 1.11–1.47). In addition, heterogeneity became nonsignificant among subgroups based on geographical area, timing of drinking, and publication year (**Table 2**).

**Table 2:** Summary results of the association between prenatal alcohol exposure and ventricular septal defects risk.

Group	No. of studies	OR(95%CI)	*P* for heterogeneity	I^2^ (%)
Total	7	1.04(0.86–1.25)	0.02	59.9
High-quality studies ^a^	5	1.13(0.95–1.33)	0.08	52.6
Geographical area				
North America	3	0.92(0.76–1.11)	0.31	15.2
Europe	3	1.06(0.80–1.41)	0.23	32.5
Australia	1	1.36(1.14–1.63	-	-
Study type				
cohort	3	1.12(0.81–1.56)	0.03	72.4
population-based case control	3	0.97(0.83–1.13)	0.93	0
hospital-based case control	1	0.69(0.35–1.36)	-	-
Timing of drinking				
first trimester	2	0.82(0.53–1.29)	0.17	47.9
during pregnancy	3	1.23(0.97–1.55)	0.16	44.0
periconception	2	0.97(0.81–1.15)	0.74	0
Publication year				
≤ 2000	4	0.91(0.77–1.06)	0.41	0
> 2000	3	1.27(1.11–1.46)	0.38	0
Sample size				
≤ 1000	2	0.91(0.66–1.28)	0.35	0
> 1000	5	1.07(0.86–1.33)	0.01	68.6

^a^ Studies scoring 6 points or higher were considered as high quality, and those scoring lower than 6 points as low quality.

#### Conotruncal defects

**Fig 4:**
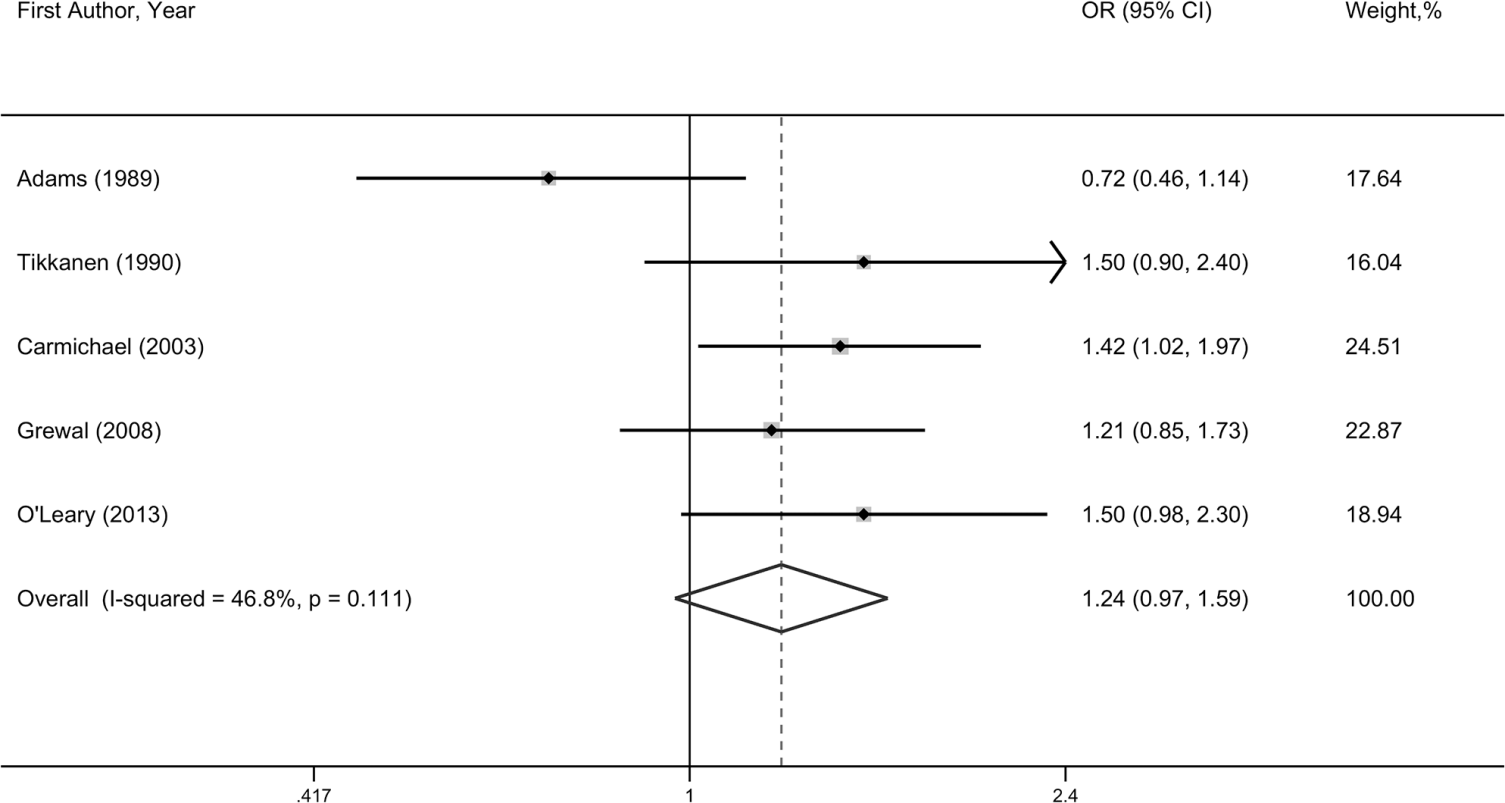
Forest plot of any prenatal alcohol exposure and conotruncal defects risk.

The association of prenatal alcohol intake with conotruncal defects (CTDs) was examined in five studies, with a total number of 716 cases and 83,825 controls. As presented in **Fig 4**, prenatal alcohol drinking was marginally significantly associated with an increased risk of CTDs (OR = 1.24, 95%CI = 0.97–1.59), and no significant heterogeneity was found (*P* = 0.11, I^2^ = 46.8%). One cohort study conducted in Australia contributed data to the association of maternal alcohol use during pregnancy with CTDs risk, and reported a borderline significant association (OR = 1.50, 95%CI = 0.98–2.30). Furthermore, subgroup analyses showed that prenatal alcohol consumption was associated with an increased risk of CTDs among studies published after 2000 (OR = 1.24, 95%CI = 1.10–1.68) and studies with large sample size (>1000) (OR = 1.35, 95%CI = 1.10–1.68) (**S2 Table**).

In addition, three studies explored the effects of prenatal alcohol exposure on the two subtypes of CTDs, Tetralogy of Fallot (TOF) and d-Transposition of the Great Arteries (dTGA). The pooled estimate showed no significant risk of TOF associated with prenatal alcohol use (OR = 1.22, 95%CI = 0.91–1.63) (**S1 Fig**). However, mothers who drank in pregnancy had 1.64 times more likely to have a newborn with d-Transposition of the Great Arteries (dTGA) (OR = 1.64, 95%CI = 1.17–2.30) (**S1 Fig**). No heterogeneity across studies was detected for the two associations (TOF: *P* = 0.48, I^2^ = 0; dTGA: *P* = 0.54, I^2^ = 0).

#### Atrial septal defects

The association between maternal alcohol exposure and atrial septal defects (ASDs) was evaluated in four studies, with a total number of 349 cases and 191,576 controls. The summarized result suggested that maternal alcohol exposure was not significantly related with ASDs risk (OR = 1.40, 95%CI = 0.88–2.23), and the heterogeneity among studies was significant (*P* = 0.02, I^2^ = 69.6%) (**S2 Fig**). When the analysis was restricted to the studies that investigated the association during the first trimester or were published before 2000, the result showed a significant association (OR = 1.83, 95%CI = 1.04–3.23). Significant result was also found in the study conducted in Australia (OR = 1.77, 95%CI = 1.27–2.46), the population-based case-control study (OR = 1.90, 95%CI = 1.03–3.50), and studies with small sample size (≤1000) (OR = 1.80, 95%CI = 1.34–2.41) (**S3 Table**). In addition, heterogeneity became negligible among studies with small and large sample size (I^2^ = 0 for both subgroups).

#### Other CHDs subtypes

No association was found in the pooled result from the two studies investigated the relationship between prenatal alcohol exposure and atrioventricular septal defects (AVSDs) (OR = 1.41, 95%CI = 0.57–3.50). Two publications by Tikkanen et al. [26, 27] reported no association of maternal alcohol drinking during the first trimester with coarctation of the aorta (COA) or Hypoplastic Left Heart Syndrome (HLHS) (COA:OR = 1.00,95%CI = 0.60–1.80; HLHS:OR = 1.69,95%CI = 0.85–3.37). Moreover, the study by O’Leary et al. [23] found no association between maternal alcohol drinking during pregnancy and the risk of Transposition of Arteries (TOA) or double outlet ventricle (TOA: OR = 1.14, 95%CI = 0.58–2.26; double outlet ventricle: OR = 1.79, 95%CI = 0.68–4.70).

#### Sensitivity analyses

Sensitivity analyses by removing of the poor-quality studies showed that the results did not change for the relationship between prenatal alcohol exposure and overall CHDs (OR = 1.11, 95%CI = 0.88–1.40), VSDs (OR = 1.13, 95%CI = 0.95–1.33), CTDs (OR = 1.24, 95%CI = 0.97–1.59), or ASDs (OR = 1.40, 95%CI = 0.84–2.35). Moreover, the association for overall CHDs or VSDs did not materially alter after exclusion of each study in turn. However, the association for CTDs turned out to be significant (OR = 1.38, 95%CI = 1.14–1.68) and the heterogeneity was greatly decreased (*P* = 0.85, I^2^ = 0%) after deletion of the study by Adams et al.[24]. Besides, the result for ASDs was converted to be significant (OR = 1.79, 95%CI = 1.34–2.38) and the heterogeneity was dramatically reduced (*P* = 0.95, I^2^ = 0%) after removal of the study by Standberg-Larser et al. [22].

#### Publication bias

**Fig 5:**
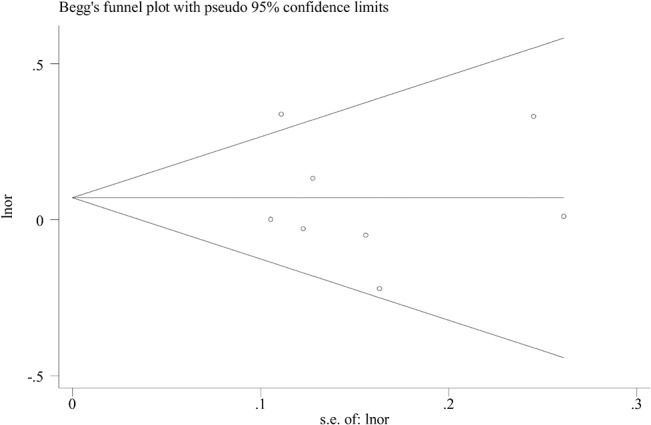
Funnel plot of any prenatal alcohol exposure and overall congenital heart defects risk.

According to Egger’s test, no publication bias was detected for the associations of prenatal alcohol consumption with overall CHDs (*P* = 0.78), VSDs (*P* = 0.12), CTDs (*P* = 0.68), TOF (*P* = 0.21), or ASDs (*P* = 0.76). **Fig 5** shows the funnel plot for the association between prenatal alcohol exposure and overall CHDs risk.

### Prenatal binge drinking vs. none drinking and CHDs risk

The effect of prenatal binge drinking on CHDs risk was assessed in a total of seven studies. In comparison with none drinking, prenatal binge drinking correlated with a 1.49 times increase in overall CHDs risk (OR = 2.49, 95%CI = 1.04–5.97) without significant heterogeneity (*P* = 0.20, I^2^ = 40.5%). However, no significant result was detected for the effects of prenatal binge drinking on the CHD subtypes (**S4 Table**).

### Dose-response relationship

There are several studies on the dose-response relationship between different levels of prenatal alcohol consumption and CHDs: four studies on alcohol consumption levels and overall CHDs[20, 21, 35, 37], three studies on VSDs[20, 22, 31], two studies on ASDs[20, 22], two studies on CTDs[30, 32], and one studies on TOF and dTGA[32]. All of these studies did not show monotonically increase or decrease in risk emerged. Because of the distinct difference in alcohol consumption levels defined in each study, we applied the classification of moderate and heavy drinking to summarize the results based on the exposure distributions in each study. The pooled results suggested that prenatal moderate drinking was associated with CTDs (OR = 1.35, 95%CI = 1.05–1.75) and dTGA (OR = 1.86, 95%CI = 1.09–3.20). Moreover, a 2.76-times increase was observed in the effects of prenatal heavy drinking on the risk of overall CHDs (OR = 3.76, 95%CI = 1.00–14.10). No significant association was found in the effects of moderate or heavy drinking on other CHDs subtypes.

## Discussion

In this meta-analysis of observational studies, we found no evidence of the association between prenatal alcohol exposure and the risk of overall CHDs, VSDs, or ASDs. However, the results suggested that prenatal alcohol drinking was marginally significantly associated with CTDs and statistically significantly associated with dTGA. Moreover, both prenatal heavy drinking and binge drinking have a strong association with overall CHDs risk, and prenatal moderate drinking has a modest association with CTDs and dTGA risk.

The nonsignificant association of prenatal alcohol exposure with overall CHDs and VSDs seemed to be stable because of the constant result in sensitivity analyses. However, the borderline significant relationship for CTDs should be treated with caution because the result turned out to be significant after removing one study. Compared with the other four included studies, this removed study reported an inverse direction of association and was conducted during the period between 1976 and 1980, which was so old that it may be the reason for the unstable result. Moreover, subgroup analysis suggested that publication year may be responsible for heterogeneity because there were distinctly different summarized results between studies published before 2000 and those after 2000. The result of no association for ASDs should also be considered cautiously because the association was converted to be significant after omitting one study. This omitted study reported a reduced risk of ASDs without statistical significance and had a large sample size of 80,148, which could influence the pooled result to a large degree. Furthermore, subgroup analysis suggested that sample size may be the source of heterogeneity because of the negligible heterogeneity among studies with small and large sample size. In addition, the significant heterogeneity for VSDs may come from geographical area, timing of drinking, and publication year according to the subgroup analysis.

Since the first publication on the fetal alcohol syndrome described by Jones and Smith in 1973[40], lots of studies have explored the relationship between maternal alcohol drinking and birth outcomes, such as birth defects, low birthweight, preterm birth, and small for gestational age. Although we could not search any other meta-analyses on the association of prenatal alcohol exposure with CHDs, we identified one meta-analysis and two systematic reviews that investigated the association with birth defects. The meta-analysis published in 1998 by Polygenis et al. [41] reported no relationship between maternal modest alcohol consumption and fetal malformations. One systematic review by Henderson et al. [13] identified six publications on the association between low-moderate prenatal alcohol exposure and congenital malformations, and only one reported a significant higher risk. In another systematic review by Henderson et al. [14], three articles that considered the association of binge drinking with congenital anomalies were included, and two found a significant increased risk. In a word, no conclusive findings could be obtained from the previous meta-analysis or systematic reviews. In contrast with these three studies, we attempted to perform the meta-analysis to study the relationship between prenatal alcohol drinking and CHDs, which are the most common group of birth defects and have great public health significance. However, the same inconclusive result was found on the association between prenatal alcohol exposure and overall CHDs. The lack of association may be partly due to the potential etiologic heterogeneity among distinct subtypes of CHDs, which may obscure the relationship when subtypes are grouped into a common phenotype to increase statistical power [42]. Additionally, according to our results, prenatal heavy drinking and binge drinking should be discouraged because of the increased risk of overall CHDs. Prenatal moderate drinking should also be noted because of the moderate higher risk of some CHDs subtypes. However, further investigation was needed due to the small number of included studies on these topics.

The methodological limitations of the included studies should be acknowledged. First, misclassification of alcohol intake may exist because no objective test or measure was performed to record the actual alcohol consumed over a period. The general volume of alcohol intake, such as one glass of wine, one bottle of beer, and one glass of spirit, was collected in many studies[20, 21, 24–28, 36], but no precise volume of alcohol consumption was considered. Various alcohol concentrations among types of alcohol used may also influence the accurate classification of alcohol use between groups. Second, alcohol use was self-reported by the participants retrospectively in the included studies, which may cause underreporting and recall bias. Underreporting may be due to the negative stigma related with alcohol drinking in pregnancy, especially when the pregnancy outcome was known[43]. Recall bias may occur because of the delay between alcohol intake and interview. The above inherent limitation of the included studies may result in non-differential or differential exposure misclassification and the influence on the actual association with CHDs is uncertain. Third, confounding bias inherent in the included studies can hardly be solved. Some crude data from the included studies were used because of the finite available information. For these data, the potential confounders, such as maternal smoking, drug use, socioeconomic status, and other environmental exposures, were not considered. Moreover, even if the adjusted data were extracted from the original studies, there was presumably still residual confounding as a result of inaccurately evaluating the confounders or not adjusting for other unmeasured ones, which may be the maternal lifestyle factors.

Other limitations of the study should also be mentioned. First, our study may be affected by selection bias. Literature searches were limited to English publications with sufficient information to access or recalculated ORs and the corresponding 95%CIs in two databases. We did not attempt to identify unpublished studies nor did we search other databases. Second, we cannot ignore possible publication bias although no evidence of publication bias was found according to the test. Studies with positive associations tended to be more easily published compared with those with negative results. However, because many clinicians have been skeptical of the association between maternal alcohol drinking and pregnancy outcome, it is also plausible that articles with negative results could be more likely to be accepted for publication [13]. Third, most of the included studies were case-control designs, which may be more vulnerable to information and selection bias. However, subgroup analyses suggested that most summarized effects of maternal alcohol drinking on CHDs were similar among different study types. Fourth, it may be questionable to extrapolate the results to the eastern countries because the included studies were all conducted in the west. Drinking patterns and the extent to which the potential exposure misclassification occur may vary across diverse countries. Moreover, the effects of alcohol intake depended on the absorption and metabolism in both mother and fetus, and the process may be partially genetically determined [17]. Thus, the relationship between maternal alcohol drinking and CHDs in infants should still be investigated in other specific groups.

Our study had several strengths. First, we obtained a large data set with a total of 310,919 mothers and 3,749 children with CHDs, which may have enough statistical power to investigate the association of prenatal alcohol intake with overall CHDs. However, the number of CHDs subtypes may be underpowered to examine the possible moderate effect of prenatal alcohol drinking on outcomes. Therefore, studies with large population are needed to be conducted and included in the meta-analysis to explore the association. Second, we were able to separately summarize a range of risks for CHDs subtypes that may be differently related with maternal alcohol consumption due to the possible heterogeneous etiologies. Third, we tried to investigate the effects of binge drinking on CHDs because animal models indicated that it was the peak blood alcohol concentration rather than the average consumption that determined the damage level[44]. Effects of moderate or heavy alcohol consumption levels on CHDs were also pooled while the dose-response analyses could not be performed because of the distinct difference in alcohol intake defined in each study. Furthermore, we applied the random-effects model to pool the risk and further conducted pre-specified subgroup analyses and sensitivity analyses to evaluate the stability and robustness of summarized results.

In conclusion, the results suggested that prenatal alcohol exposure was not associated with overall CHDs or some subtypes, whereas marginally significant association was found for CTDs and statistically significant association was found for dTGA. Given the limitations of our study, further prospective studies with large population and better study designs, such as accurate classification of alcohol exposure (amount, duration, frequency, and timing of alcohol intake), adequate control for important confounders, are needed to explore the actual relationship between prenatal alcohol drinking and CHDs, especially with regard to the CHDs subtypes. Moreover, more studies, particularly in the eastern countries, are still needed to investigate the association of prenatal alcohol exposure with CHDs including the subtypes in specific groups.

## Supporting Information

S1 PRISMA Checklist PRISMA Checklist. (DOC)

S1 Fig Forest plot of prenatal alcohol exposure and the two subtypes of conotruncal defects risks. (TIF)

S2 Fig Forest plot of prenatal alcohol exposure and atrial septal defects risk. (TIF)

S1 Table Characteristics of the included studies on the association of prenatal alcohol exposure with congenital heart defects risk. (DOC)

S2 Table Summary results of the association between prenatal alcohol exposure and conotruncal defects risk. (DOC)

S3 Table Summary results of the association between prenatal alcohol exposure and atrial septal defects risk. (DOC)

S4 Table Summary results of the association between prenatal binge drinking and CHDs risk. (DOC)
