# Supplementary figures and images for: Prenatal Alcohol Exposure and Congenital Heart Defects: A Meta-Analysis
Source: PLoS One. 2015 Jun 25;10(6):e0130681. doi: 10.1371/journal.pone.0130681 (PMC4482023; doi:10.1371/journal.pone.0130681)

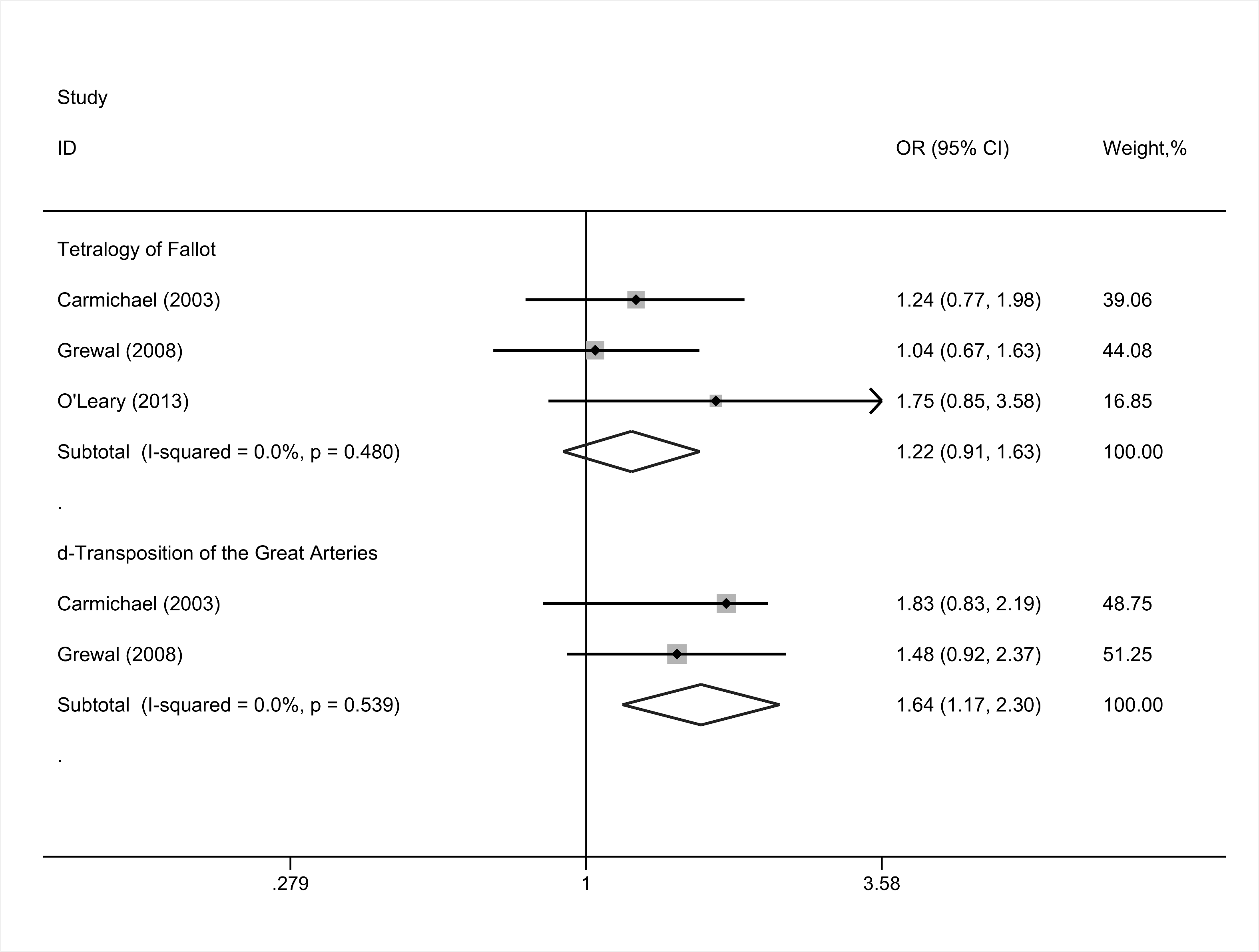

Supplement: S1 Fig — (TIF) [file pone.0130681.s002.tif]

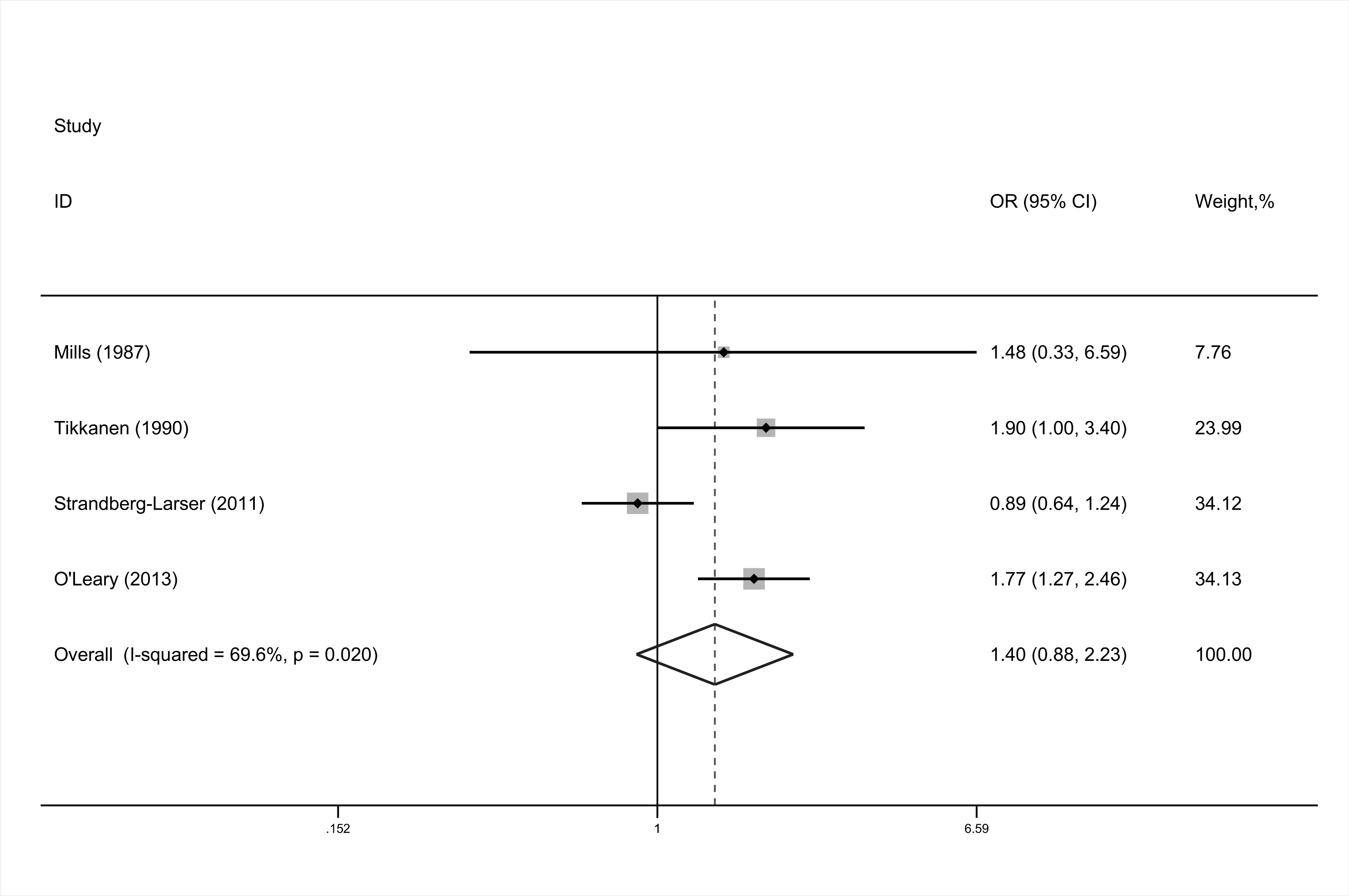

Supplement: S2 Fig — (TIF) [file pone.0130681.s003.tif]
