# Supplementary material for: Prenatal Alcohol Exposure and Congenital Heart Defects: A Meta-Analysis
Source: PLoS One. 2015 Jun 25;10(6):e0130681. doi: 10.1371/journal.pone.0130681 (PMC4482023; doi:10.1371/journal.pone.0130681)
Supplement: S1 Table — (DOC) [file pone.0130681.s004.doc]

| **S1 Table.** Characteristics of the included studies on the association of prenatal alcohol exposure with congenital heart defects risk. | | | | | | | | | | |
| --- | --- | --- | --- | --- | --- | --- | --- | --- | --- | --- |
| **first author** | **publication  year** | **study period** | **study country** | **study type** | **sample  size** | **ascertainment of drinking exposure** | **ascertainment of CHDs** | **timing of drinking** | **NOS score** | **matched or adjusted confounders** |
| Mills | 1987 | 1974-1977 | America | cohort | 30286 | in-person interview | medical records | T1 | 5 | none |
| Adams | 1989 | 1976-1980 | America | PCC | 1352 | telephone interview | medical records | P | 8 | birth quarter, hospital |
| Tikkanen | 1990 | 1982-1983 | Finland | PCC | 1164 | in-person interview | confirmed by C/E/S/A | T1 | 8 | maternal age, smoking,  coffee, organic solvents  and so on |
| McDonald | 1992 | 1982-1984 | Canada | cohort | 87727 | in-person interview | medical records | T1 | 4 | none |
| Tikkanen | 1993 | 1982-1983 | Finland | PCC | 806 | in-person interview | confirmed by C/E/S/A | T1 | 8 | maternal age, smoking,  coffee, organic solvents  and so on |
| Tikkanen | 1994 | 1982-1983 | Finland | PCC | 790 | in-person interview | confirmed by C/E/S/A | T1 | 6 | none |
| Ewing | 1997 | 1981-1989 | America | PCC | 4040 | in-person interview | confirmed by C/E/S/A | P | 6 | none |
| Sands | 1999 | 1994-1998 | Ireland | HCC | 312 | in-person interview | confirmed by E | D | 4 | none |
| Cedergren | 2002 | 1982-1996 | Sweden | PCC | 748 | medical records | medical records | T1 | 5 | maternal age,  birth year |
| Carmichael | 2003 | 1987-1988 | America | PCC | 685 | telephone interview | confirmed by C/E/S/A | P | 6 | gender, birth place |
| Martinez-Frias | 2004 | 1977-2001 | Spain | HCC | 3389 | in-person interview | CE and E | D | 7 | maternal smoking, race, multivitamin use, education |
| Williams | 2004 | 1968-1980 | America | PCC | 3143 | telephone interview | medical records | P | 7 | birth year, race, birth period,  birth hospital |
| Grewal | 2008 | 1999-2004 | America | PCC | 681 | telephone interview | confirmed by C/E/S/A | M1 | 6 | none |
| Kuciene | 2009 | 1990-2005 | Lithuania | PCC | 814 | in-person or  telephone interview | medical records | D | 4 | none |
| Cresci | 2011 | 2008-2010 | Italy | HCC | 720 | in-person interview | medical records | P | 4 | none |
| Hobbs | 2011 | 1998-2008 | America | HCC | 664 | in-person interview | confirmed by C/E/S/A | P | 4 | none |
| Strandberg-Larsen | 2011 | 1996-2002 | Denmark | cohort | 80346 | in-person interview | medical records | D | 8 | maternal age, smoking, parity, occupational status |
| Patel | 2012 | 1997-2005 | America | PCC | 6866 | telephone interview | confirmed by C/E/S/A | P | 7 | birth year,  birth site |
| Mateja | 2012 | 1996-2005 | America | PCC | 1157 | by email or telephone interview | birth certificate | B3 | 5 | none |
| O'Leary | 2013 | 1983-2007 | Australia | cohort | 85229 | birth records | birth defects registers | D | 7 | maternal age,  race, birth year |
| PCC: population-based case-control study; HCC: hospital-based case-control study; C: cardiac catheterization; E: echocardiography;  S: cardiac surgery; A: autopsy; CE: clinical examination; P: periconception; T1: first trimester; D:during pregnancy;  B3: three months before pregnancy; M1: first month after pregnancy. | | | | | | | | | | |
