# Supplementary material for: Prenatal Alcohol Exposure and Congenital Heart Defects: A Meta-Analysis
Source: PLoS One. 2015 Jun 25;10(6):e0130681. doi: 10.1371/journal.pone.0130681 (PMC4482023; doi:10.1371/journal.pone.0130681)
Supplement: S2 Table — (DOC) [file pone.0130681.s005.doc]

| **S2 Table.** Summary results of the association between prenatal alcohol exposure and conotruncal defects risk. | | | | |
| --- | --- | --- | --- | --- |
| **Group** | **No. of studies** | **OR(95%CI)** | ***P* for heterogeneity** | **I^2^ (%)** |
| Total | 5 | 1.24(0.97-1.59) | 0.11 | 46.8 |
| High-quality studies ^a^ | 5 | 1.24(0.97-1.59) | 0.11 | 46.8 |
| Geographical area |  |  |  |  |
| North America | 3 | 1.10(0.76-1.60) | 0.06 | 65.2 |
| Europe | 1 | 1.50(0.92-2.45) |  |  |
| Australia | 1 | 1.50(0.98-2.30) |  |  |
| Study type |  |  |  |  |
| cohort | 1 | 1.50(0.98-2.30) |  |  |
| population-based case control | 4 | 1.18(0.88-1.59) | 0.08 | 55 |
| Timing of drinking |  |  |  |  |
| first trimester | 1 | 1.50(0.90-2.40) |  |  |
| during pregnancy | 1 | 1.50(0.98-2.30) |  |  |
| periconception | 2 | 1.03(0.53-2.00) | 0.02 | 82.3 |
| first month | 1 | 1.21(0.85-1.73) |  |  |
| Publication year |  |  |  |  |
| ≤ 2000 | 2 | 1.03(0.50-2.12) | 0.03 | 78.4 |
| >2000 | 3 | 1.24(1.10-1.68) | 0.71 | 0 |
| Sample size |  |  |  |  |
| ≤ 1000 | 2 | 1.04(0.51-2.14) | 0.02 | 81.3 |
| > 1000 | 3 | 1.35(1.10-1.68) | 0.73 | 0 |
| ^a^ Studies scoring 6 points or higher were considered as high quality, and those scoring lower than 6 points as low quality. | | | | |
|  | | | | |
