# Supplementary material for: Prenatal Alcohol Exposure and Congenital Heart Defects: A Meta-Analysis
Source: PLoS One. 2015 Jun 25;10(6):e0130681. doi: 10.1371/journal.pone.0130681 (PMC4482023; doi:10.1371/journal.pone.0130681)
Supplement: S3 Table — (DOC) [file pone.0130681.s006.doc]

| **S3 Table.** Summary results of the association between prenatal alcohol exposure and atrial septal defects risk. | | | | |
| --- | --- | --- | --- | --- |
| **Group** | **No. of studies** | **OR(95%CI)** | ***P* for heterogeneity** | **I^2^ (%)** |
| Total | 4 | 1.40(0.88-2.23) | 0.02 | 69.6 |
| High-quality studies ^a^ | 3 | 1.40(0.84-2.35) | 0.007 | 79.7 |
| Geographical area |  |  |  |  |
| North America | 1 | 1.48(0.33-6.61) |  |  |
| Europe | 2 | 1.24(0.59-2.60) | 0.03 | 78.1 |
| Australia | 1 | 1.77(1.27-2.46) |  |  |
| Study type |  |  |  |  |
| cohort | 3 | 1.28(0.72-2.28) | 0.02 | 76 |
| population-based case control | 1 | 1.90(1.03-3.50) |  |  |
| Timing of drinking |  |  |  |  |
| first trimester | 2 | 1.83(1.04-3.23) | 0.76 | 0 |
| during pregnancy | 2 | 1.26(0.64-2.46) | 0.004 | 88 |
| Publication year |  |  |  |  |
| ≤ 2000 | 2 | 1.83(1.04-3.23) | 0.76 | 0 |
| > 2000 | 2 | 1.26(0.64-2.46) | 0.004 | 88 |
| Sample size |  |  |  |  |
| ≤ 1000 | 2 | 1.80(1.34-2.41) | 0.84 | 0 |
| > 1000 | 2 | 0.91(0.66-1.26) | 0.52 | 0 |
| ^a^ Studies scoring 6 points or higher were considered as high quality, and those scoring lower than 6 points as low quality. | | | | |
|  | | | | |
