# Supplementary material for: Prenatal Alcohol Exposure and Congenital Heart Defects: A Meta-Analysis
Source: PLoS One. 2015 Jun 25;10(6):e0130681. doi: 10.1371/journal.pone.0130681 (PMC4482023; doi:10.1371/journal.pone.0130681)
Supplement: S4 Table — (DOC) [file pone.0130681.s007.doc]

| **S4 Table.** Summary results of the association between prenatal binge drinking and CHDs risk. | | | | | |
| --- | --- | --- | --- | --- | --- |
| outcomes | No. of  cases/controls | No. of studies | OR(95%CI) | *P* for heterogeneity | I^2^ (%) |
| overall CHDs | 2375/4116 | 2 | 2.49(1.04-5.97) | 0.20 | 40.5 |
| VSDs | 443/81848 | 2 | 1.10(0.85-1.41) | 0.39 | 0 |
| CTDs | 485/2195 | 3 | 1.10(0.59-2.08) | 0.17 | 43.6 |
| TOF | 199/928 | 2 | 0.86(0.43-1.73) | 0.13 | 55.8 |
| dTGA | 159/928 | 2 | 1.98(0.82-4.80) | 0.9 | 0 |
| ASDs | 145/80183 | 1 | 0.80(0.54-1.18) | - | - |
| CHDs: congenital heart defects; VSDs: ventricular septal defects; ASDs: atrial septal defects; CTDs: conotruncal defects; TOF: Tetralogy of Fallot;  dTGA: D-transposition of the Great Arteries. | | | | | |
